# A systematic review of personality disorder, race and ethnicity: prevalence, aetiology and treatment

**DOI:** 10.1186/1471-244X-10-33

**Published:** 2010-05-11

**Authors:** Angela McGilloway, Ruth E Hall, Tennyson Lee, Kamaldeep S Bhui

**Affiliations:** 1 Barts and The London School of Medicine and Dentistry, Turner Street, London E1 2AD, UK; 2 Centre for Psychiatry, Barts & The London School of Medicine & Dentistry, Old Anatomy Building, Charterhouse Square, London, EC1M 6BQ, UK; 3 The Centre for Applied Research and Evaluation International Foundation (Careif), Centre for Psychiatry, Barts & The London School of Medicine & Dentistry, Old Anatomy Building, Charterhouse Square, London, EC1M 6BQ, UK; 4 East London Foundation Trust, Trust headquarters, Eastone, 22 Commercial Street, London, E1 6LP, UK

## Abstract

**Background:**

Although psychoses and ethnicity are well researched, the importance of culture, race and ethnicity has been overlooked in Personality Disorders (PD) research. This study aimed to review the published literature on ethnic variations of prevalence, aetiology and treatment of PD.

**Method:**

A systematic review of studies of PD and race, culture and ethnicity including a narrative synthesis of observational data and meta-analyses of prevalence data with tests for heterogeneity.

**Results:**

There were few studies with original data on personality disorder and ethnicity. Studies varied in their classification of ethnic group, and few studies defined a specific type of personality disorder. Overall, meta-analyses revealed significant differences in prevalence between black and white groups (OR 0.476, CIs 0.248 - 0.915, p = 0.026) but no differences between Asian or Hispanic groups compared with white groups. Meta-regression analyses found that heterogeneity was explained by some study characteristics: a lower prevalence of PD was reported among black compared with white patients in UK studies, studies using case-note diagnoses rather than structured diagnostic interviews, studies of borderline PD compared with the other PD, studies in secure and inpatient compared with community settings, and among subjects with co-morbid disorders compared to the rest. The evidence base on aetiology and treatment was small.

**Conclusion:**

There is some evidence of ethnic variations in prevalence of personality disorder but methodological characteristics are likely to account for some of the variation. The findings may indicate neglect of PD diagnosis among ethnic groups, or a true lower prevalence amongst black patients. Further studies are required using more precise cultural and ethnic groups.

## Background

Personality Disorder (PD) is defined by the World Health Organisation as "*a severe disturbance in the characterological condition and behavioural tendencies of the individual, usually involving several areas of the personality, and nearly always associated with considerable personal and social disruption*"[1].

The nature, diagnosis and categorisation of PD has been widely deliberated among mental health professionals, yet has been subjected to little empirical research [2]. Nonetheless, a good deal of information is known regarding PD [3]. One aspect that has been overlooked that may reveal a better understanding about the aetiology and treatment of personality disorder is the impact of culture, race and ethnicity on PD [2]. Black and minority ethnic groups are known to be over-represented in mental health services, especially in forensic and secure settings and inpatient care. Similar studies of PD are uncommon. PD research is fraught with problems. The category of PD has been criticised as culturally biased [4] and that the diagnosis is a reflection of North American and Western European concepts of personality functioning [5]. Behavioural norms in one culture may be considered deviant in another, however, there are insufficient studies addressing the role of ethnicity in diagnostic practice [5]. This study aimed to systematically review all available published literature that addresses PD prevalence, aetiology and treatment in relation to race and ethnicity.

## Method

We searched PUBMED, EMBASE, CINAHL, PsycINFO and Web of Science for studies relating to PD and race, culture and ethnicity. Searches were undertaken between the 26^th ^February and the 7^th ^of March 2008. Inclusion criteria were set widely for studies with original data on race and ethnic group, with personality disorder as an outcome. The subjects of the studies were adults and the settings included community, specialist mental health services and prison settings. The search was supplemented by forward and backward citation, manual exploration of references and by contacting experts in the field to refer us to any other relevant studies.

**Figure 1:**
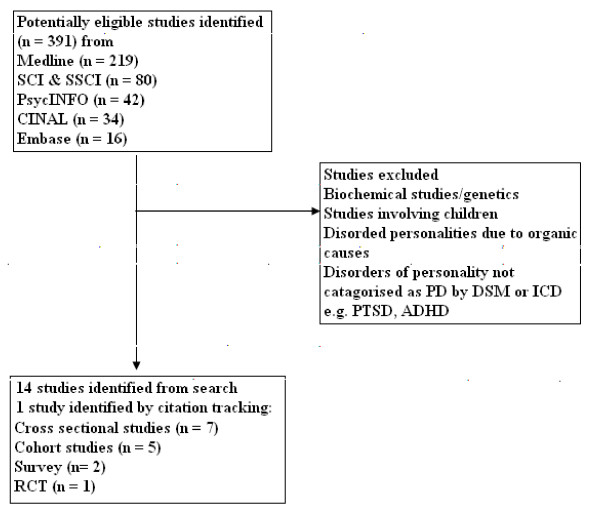
**QUOROM flow chart of studies in the review**.

Of the 391 publications identified by the search, after review of full text articles, fourteen studies met the inclusion criteria for the review. Reference tracking identified one further study resulting in a total of fifteen studies for review (see Figure 1).

From the 15 publications (13 studies) entering the review, the following data were extracted and tabulated (Tables 1 &2): outcome of interest (prevalence, aetiology, and treatment), description of methods used (study design, procedure, diagnostic tool, statistical methods), participants, place of study (country and setting), main effects and data points for our outcomes of interest, and strengths and limitations of each study. In addition to these, a scoring system for the methodological quality was designed by one reviewer (AM), and adapted with a second reviewer (KB) experienced in systematic review methods in order. Six domains were considered (see Table 3).

**Table 1:** Study characteristics

Author	Objective	Study Design	Procedure	Inclusion/exclusion
Mikton C. Grounds A. 2007	Examine cross-cultural clinical judgement bias in the diagnosis of PD in Afro-Caribbean men	Two vignettes of male patients, Afro-Caribbean or white, one suggestive of BPD the other suggestive of ASPD sent to psychiatrists. Participants chose diagnosis from list.	2 vignettes sent to each psychiatrist.	All consultants and specialist registrars in forensic psychiatry in the UK included.

Al-Saffar S. Borga P. Wicks S. Hallstrom T. 2004	Describe the distribution of different ethnic patient groups in Psych OPD and influence of ethnicity, on diagnosis.	Retrospective cohort study using outpatients documentation	Exploration of register for ethnicity and diagnosis	Patients over 18 years of age

Castaneda R. Franco H. 1985	Examine sex and ethnic distribution of BPD in a psychiatric inpatient sample	Retrospective study of 1,583 inpatients discharged in index year using patient notes.	Patients' charts reviewed, primary psychiatric diagnosis and demographics extracted.	Patients with co-existing axis I disorder diagnosis excluded.

Tyrer P. Merson S. Onyett S. Johnson T. 1994	To compare community-based and standard hospital psychiatric services, including PD as an outcome.	RCT of community EIS vs conventional hospital psychiatric services over 14 months for psychiatric emergency patients.	Pt assessed for PD before being randomly assigned to either treatment setting for 12 weeks	Age 16-65. No alcohol/drug dependence. No mandatory care necessary. Not in contact with psych services.

Trestman RL. Ford J. Zhang W. Wiesbrock V. 2007	To estimate percentage of undiagnosed prison inmates who meet diagnostic criteria for psychiatric illness.	Newly admitted patients in 5 prisons assessed for psychiatric illness.	All participants interviewed once for screening. Random sample further interviewed by 5 trained assessors	Excluded: under 18, high bonds, those in security restricted housing, already under medical/mental health care

Maden A. Friendship T. McClintock T. Rutter S. 1999	To test the hypothesis that there are systematic differences in clinical outcome in patients of different ethnic origin.	Longitudinal cohort study of discharges from a medium secure unit (average follow up 6.6 yrs)	Admission & short term data from MDT records. Long term info from all med records, Home Office Register, Prison records, Offenders index, NHS central record, Special Hospitals case register, & semi-structured interviews	All patients discharged from a first admission to The Denis Hill Unit of the Bethlem Royal Hospital from Oct 1980 till Oct 1994

Coid J. Petruckevitch A. Bebbington P. Brugha T. Bhugra D. et al 2002	To estimate population-based rates of imprisonment in different ethnic groups, & compare criminal behaviour & psychiatric morbidity	Examination of home office data on all inmates, and cross-sectional survey of remanded and sentenced prisoners in 1997	Survey comprised lay interviews/self administered, then every 5th participant had follow-up interview by clinician	All prisoners on remand or sentenced in England & Wales in 1997 included.

Coid J. Petruckevitch A. Bebbington P. Brugha T. Bhugra D. et al 2002	To compare early environmental risks, stressful daily living experiences & reported use of psych services in prisoners from diff ethnic grps	Examination of home office data on all inmates, and cross-sectional survey of remanded and sentenced prisoners in 1997	Survey comprised lay interviews/self administered, then every 5th participant had follow-up interview by clinician	All prisoners on remand or sentenced in England & Wales in 1997 included.

Coid J. Kahtan N. Gault S. Jarman B. 2000	To estimate population-based prevalence rates of treated mental disorder in different ethnic groups compulsorily admitted to secure forensic psychiatry services	Retrospective survey of 3155 first admissions from 1988 to 1994 from half of England and Wales with 1991 census data as the denominator adjusted for under-enumeration	Item sheets completed from case notes. Data collected by clinically trained research psychiatrist	Those with no fixed abode excluded

Coid J. Kahtan N. Gault S. Jarman B. 1999	To compare patients with PD and mental illness according to demography, referral, criminality, previous institutionalisation and diagnostic comorbidity	Retrospective survey of all admissions from 1988 to 1994 from 7 (of 14) regional health authority catchment areas in England & Wales	One researcher completed item sheet for every admission. recorded demography, nature of referral, legal status & catchment of origin	All admissions of pts with PD to special hospitals and MSU from a geographically representative area

Bender DS. Skodol AE. Dyck IR. Markowitz JC. Shea MT. et al 2007	To explore whether PD psychopathology raises particular challenges to treatment-seeking ethnic minorities receiving adequate mental health services	2 year prospective study: of patients recently treated or seeking treatment from clinical services. Follow up at 6, 12, 24 months.	Experienced research clinicians determined 1 of 4 PD Δ: Schizotypal (STPD), BPD, Avoidant (AVPD) & Obsessive-compulsive (OCPD) by interview	Treatment-seeking/recently treated pts 18-45. Exclusion: active psychosis, acute substance intoxication/withdrawalhistory of schizophrenia/schizoaffective/schizophreniform disorders

Chavira DA. Grilo CM. Shea T. Yen S. Gunderson JG. et al 2003	Compare the relative proportion of 4 PDs among 3 ethnic grps in a clinical sample & examine whether specific PD criteria accounted for difference in ethnic distribution	Survey/Questionnaire. Patients filled out Personality Screening Questionnaire: If +ve for 1 or more PDs they were referred for further assessment. Also completed Depression Screening Questionnaire: If +ve were referred as potential controls	Patients interviewed by trained & experienced interviewers using DSM-IV & Personality Assessment form. Patients also asked to fill in self-report questions. If DSM-IV supported by any instrument, patients were assigned to PD	Treatment-seeking/recently treated patients, aged 18-45. Exclusion: active psychosis, acute substance intoxication/withdrawal, history of schizophrenia/schizoaffective/schizophreniform disorders

Iwamasa GY. Larrabee AL. Merritt RD. 2000	Assess possible ethnicity criterion bias of DSM-III-R PDs using a lay sample of college undergraduates with no previous education on psychological disorders	Random card-based task with personality characteristics to be sorted by participants' own beliefs not stereotypes.	Participants sorted cards 3 separate times by ethnicity	College students unfamiliar with DSM-III-R excluded

Huang B. Grant BF. Dawson DA. Stinson FS. Chou SP. Et al 2006	Compare the current prevalence & co-occurrence of DSM-IV, alcohol & drug use disorders & mood, anxiety & PDs among whites, blacks, Native Americans, Asians & Hispanics in a large representative sample of the US population	Face-to-face survey of 43093 participants by National Epidemiological Survey on Alcohol and Related Conditions (NESARC).	Interview administered using laptop computer-assisted software. Used professional interviewers from US Bureau	Civilian non-institutionalised respondents aged 18+.

Compton WM. Cottler LB. Abdallah AR. Phelps DL. Spitznagel EL. & Horton JC. 2000	Determine the rates of specific psychiatric disorders among drug dependent persons in treatment and determine whether these rates vary by race (and gender)	Interview-based study of newly admitted patients. Two face-to-face interview sessions 12 months apart.	Subjects randomly selected from lists of newly admitted pts from the data from a longitudinal study of substance abusers 1^st^	Substance abusers who were recently admitted to drug treatment facilities in St Louis.

PD: Personality Disorder

RCT: Randomised Control Trial

EIS: Early intervention Service

MSU: Medium Secure Unit

**Table 2:** Study results

Author	Results	Prevalence
Mikton C. Grounds A. 2007	Vignette 1 (BPD): no sig diff in diagnosis PD. Vignette 2 (ASPD): More Caucasian than afro-Caribbean diagnosed ASPD (OR 2.6, 95% CI 1.5-4.4, p = 0.0006) or with any PD (OR 2.7, 1.6-4.7, p = 0.0002). Clinicians 2.8 (1.6-5.0 p < 0.001) times more likely to attribute any PD to Caucasian than afro-Caribbean. Non-white clinicians are 2.2 (1.1-4.6 p = 0.04) times more likely than white clinicians to attribute a diagnosis of any PD to vignette II	Not real pts - hypothetical examples

Al-Saffar S. Borga P. Wicks S. Hallstrom T. 2004	PD related to Swedish origin OR 2.16, CI 1.51-3.09, p = 0.05.	

Castaneda R. Franco H. 1985	Females at least 3 times more likely than males to have BPD, except in Hispanic population where no diff found. Black: t = 2.57 df 23 p < 0.02. White: t = 2.72 df 39 p < 0.01. More Hispanic men were diagnosed with BPD than white or black men (x2 = 4.39, df 1, p < 0.05). No sig diff among females of diff ethnic grps. No sig diff among ethnic grps overall	101/1583 inpatient sample had PD: White 41/101 (40.6%) Black 25/101 (24.8%) Hispanic 34/101 (33.7%) Other 1/101 (0.9%) In each population: White 41/577 (7.1%) Black 25/558 (4.5%) Hispanic 34/402 (8.5%) Other 1/46 (2.2%)

Tyrer P. Merson S. Onyett S. Johnson T. 1994	63% Caucasian patients diagnosed with PD compared to only 25% of other races (mostly Afro-Caribbean) x2 12.4, df 1, p < 0.001 OR 0.2 (0.07-0.6)	63% Caucasian patients diagnosed with PD compared to only 25% of other races (mostly Afro-Caribbean) x2 = 12.4, df 1, p < 0.001 OR 0.2 (0.07-0.6)

Trestman RL. Ford J. Zhang W. Wiesbrock V. 2007	No significant differences between race in ASPD or BPD. Hispanic men (56.7%) were more likely to meet the criteria for Cluster B diagnosis than white (39.7%) or black (37.7%) men (x2 = 7.18, 2 df, p < 0.05) Hispanic men more likely to ASPD (53.7%) than white (35.7%) or black (35.5%) (x2 = 7.18, 2 df, p < 0.05)	Axis II disorder: White 5.1% (12/218) Black 5.7% (10/177) Hispanic 11% (12/110) ASPD: White 30.7% Black 32.4% Hispanic 45.9% BPD: White 20.3% Black 11.6% Hispanic 17.4%

Maden A. Friendship T. McClintock T. Rutter S. 1999	White patients had a higher incidence of PD compared to black patients (22% vs 6% OR = 4.52 95% CI 1.79-11.4 no p value given, although discussed as statistically significant)	In ethnic pop: White 28/125 (22% of white pop) Black 6/100 (6% of black pop) With PD: White 28/34 (82.4%) Black 6/34 (17.6%) In sample: White 28/225 (12.4%) Black 6/225 (2.7%) Overall 34/225 (15.1%)

Coid J. Petruckevitch A. Bebbington P. Brugha T. Bhugra D. et al 2002	For any PD, black men had a lower risk than white men in unadjusted analyses: OR 0.67 (0.51-0.88) p = 0.004. These findings are not sustained in adjusted analyses. South Asian men similarly had a lower risk than whites (OR 0.54 (0.33-0.87) p = 0.012) respectively. Conversely, more women prisoners received a diagnosis of PD than white females (adjusted OR 2.31 (1.27-4.2) p = 0.006)	Raw figures not provided, only calculated ORs

Coid J. Petruckevitch A. Bebbington P. Brugha T. Bhugra D. et al 2002	Black people with PD less likely to have had prior treatment than white people. White pop more likely to have PD: Black men OR 0.49 (0.27-0.9) p = 0.022 Black women OR 0.13 (0.05-0.34) p < 0.001. White women were more likely to have the following PDs compared with black women: OCD, Paranoid, Schizotypal, BPD and Antisocial PD	Raw figures not provided, only calculated ORs

Coid J. Kahtan N. Gault S. Jarman B. 2000	For any PD, black patients had less risk than whites (OR 0.22 (0.15-0.31) p < 0.001), Asians also had lower risk OR 0.1 (0.03-0.41) [p < 0.001]	In ethnic pop: White 452/2224 (20%) Black 33/628 (5%) Asian 2/80 (3%) With PD: White 452/487 (92.8%) Black 33/487 (6.8%) Asian 2/487 (0.4%) Entire sample: White 452/2932 (15.4%) Black 33/2932 (0.01%) Asian 2/2932 (0.06%)

Coid J. Kahtan N. Gault S. Jarman B. 1999	Patients w PD more likely to be Caucasian (470/511 92%) than were those with mental illness (1833/2575 71%) OR 4.62, 3.32-6.43 p < 0.001. Afro-Caribbean mentally ill (615/2575 24%) compared w PD (33/511 6%) OR 4.55, 3.16-6.55 p < 0.001. Pts w PD more likely to be UK-born than those w mental illness (488 95% vs 2137 83%) OR 4.34, 2.82-6.68 p < 0.001	With PD: White 470/511 (92%) Afro-Caribbean 33/511 (6%)

Bender DS. Skodol AE. Dyck IR. Markowitz JC. Shea MT. et al 2007	Baseline data: African American (OR 0.22, 0.07-0.7) & Hispanic (OR 0.47, 0.09-0.96) less likely to received psychosocial Rx of any type in lifetime compared to white p = 0.0206, or received psychotropic med (AA OR 0.35, 0.02-0.71. His OR 0.37, 0.16-0.83. p < 0.01) & White pts w BPD more wks psychiatric hospitalisation p = 0.01	With PD: White 396/548 (72.3%) African American 78/548 (14.2%) Hispanic 74/548 (13.5%)

Chavira DA. Grilo CM. Shea T. Yen S. Gunderson JG. et al 2003	Hispanics had disproportionately more BPD than Caucasians (p < 0.001) and African Americans (p < 0.01). For STPD, African Americans had disproportionately more diagnoses than Caucasians (p < 0.05 and Hispanics (p < 0.05. No sig diff for AVPD or OCPD	With PD: 433/554 White (78.2%) 65/554 African American (11.7%) 56/554 Hispanic (10.1%)

Iwamasa GY. Larrabee AL. Merritt RD. 2000	Results suggest PD criteria were distributed systematically such that PD diagnosis were applied to certain ethnic grps. African American given Antisocial & paranoid PDs. Schizoid PD applied to Asian Americans. Schizotypal PD applied to Native Americans. All other PDs were applied to European Americans (BPD, Dependant, Narcissistic, & Obsessive-Compulsive). All p < 0.001.	Not real pts - hypothetical examples

Huang B. Grant BF. Dawson DA. Stinson FS. Chou SP. Et al 2006	Native Americans had the highest prevalence of PD, and Asians the lowest (see prevalence). Association between PD and Alcohol and Drug were positive & sig (except for Drugs & PD in Asians). This is true of unadjusted and adjusted (for age, income marital status, religion, sex, & urban city) ORs. Associations btwn alcohol & PD (1.7-5.0) were generally lower than between drugs & PD (2.1-6.3)	Prevalence captured in weighted % White 14.6% Black 16.6% (significant differences compared with White p < 0.05) Native American 24.1% (significant differences when White & black were compared, at p < 0.05). Asian 10.1% (significantly different from White, Black & N. Americans, at p < 0.05). Hispanic 14% (significantly different from other 4 ethnicities p < 0.05)

Compton WM. Cottler LB. Abdallah AR. Phelps DL. Spitznagel EL. & Horton JC. 2000	Antisocial PD present in 44% of respondents with drug dependence: 49% African American males, 26% African American females. 52% White males, 39% White females. The difference between race and PD w drug dependence was not sig. (i.e. p > 0.05). However, White race was associated with higher rates of generalised anxiety disorder than African Americans (p < 0.05) 6% African American men vs 15% White men & 7% African American women vs 16% White women	Antisocial PD within ethnic pop: 109/258 African American (42%) 77/167 Caucasian (46%) Antisocial PD: African American 109/186 (58.6%) Caucasian 77/186 (41.4%) Total sample: African American 109/425 (25.6%) Caucasian 77/425 (18.1%)

**Table 3:** Scoring system for quality of included papers

Sample of patients	Sample size	Definition & diagnosis of PD	Breakdown of ethnicity	Data Collection	Discussion & analysis	Scoring
Not specified	< 30	None	2 divisions only	2^nd^/3^rd ^party report collection	No attempt to explain findings	0
Specific group e.g. prisoners	≥ 30	Appropriate tool by non-clinician	More than 2 divisions	First hand collection	Explanation for findings offered	1
General Population	Considered e.g. power calculation	Appropriate tool by clinician				2

(QUALITY: 0-3; low, 4-6; moderate, 7-9; high)

The studies differed in methods and objectives. Therefore, the observational data were subjected to a narrative synthesis in order to identify common and recurring themes from different papers[6] Of the fifteen papers, seven provided raw prevalence data by ethnic group that could be used in a meta-analysis (additional file 1). The software package Comprehensive Meta Analysis (version 2) was used to calculate odds ratios for PD in an ethnic compared to white group. Heterogeneity was calculated using I^2 ^as this is more useful than Cochran's Q value in showing the extent of heterogeneity in small samples [7]. A value of zero reflects true homogeneity amongst studies whilst values above this show the presence of heterogeneity. Values around I^2 ^= 25, 50 and 75 reflect low, moderate and high heterogeneity respectively[7]. Where I^2 ^exceeded 75, a random effects model was used, below this level a fixed effects model was used.

In order to further explore possible causes of between-study heterogeneity, meta-regression analyses were performed (see Table 4). These compared black with white groups by the following characteristics: US and UK studies; community, inpatient and prison settings; secure and non-secure inpatient settings; use of an interview schedule and no interview schedule; different diagnoses (antisocial personality disorder, borderline personality disorder and both combined); and personality disorder diagnosis alone and with co-morbidity. Age and gender of participants were not extracted as only three studies provided this data.

**Table 4:** Results of analyses looking at sources of heterogeneity

*Study characteristics*	*No. of studies*	*Odds Ratio of PD in black compared to white groups* *(95% CI)*	*Heterogeneity (I^2^)*
Geographical area: US	4^2378^	0.872 (0.634 - 1.199)	74.925
Geographical area: UK	3^145^	0.214 (0.167 - 0.274)	0.00

Clinical setting: health service	5^1-5^	0.357 (0.188 - 0.677)	89.919
Clinical setting: secure inpatient	3^145^	0.214 (0.167 - 0.274)	0.00
Clinical setting: non-secure health service	2^23^	0.755 (0.551 - 1.035)	2.201
Clinical setting: prison	1^7^	0.759 (0.510 - 1.131)	0.00
Clinical setting: community	1^8^	1.164 (1.087 - 1.245)	0.00

Interview schedule	3^278^	1.140 (1.067 - 1.218) fixed effects	68.815
No interview schedule	4^13-5^	0.281 (0.169 - 0.467) random effects	77.274

Diagnosis: ASPD	2^27^	0.948 (0.710 - 1.265)	0.00
Diagnosis: BPD	2^37^	0.575 (0.394 - 0.840)	0.00
Diagnosis: ASPD and BPD	2^47^	0.405 (0.119 - 1.381)	95.140

Co-morbidity	5^12457^	0.381 (0.190 - 0.764)	92.288
No co-morbidity	3^378^	0.789 (0.432 - 1.441)	76.81

## Results

Of the 15 studies reviewed, 9 were of moderate quality and 5 of high quality. Studies included surveys, cohorts, cross-sectional and randomised controlled trials, and took place in a variety of environments including civilian populations, prisons, forensic units, psychiatric emergency clinics, and both inpatient and outpatient settings; studies were equally from the US and the UK.

### Defining PD

Interview schedules were used to establish PD prevalence in three studies; the schedules included the NIMH Diagnostic Interview Schedule Version III-R [8], the Alcohol Use Disorder and Associated Disabilities Interview Schedule-DSM IV version [9], the Structured Clinical Interview for DSM-IV Axis II [10], and the Structured Clinical Interview for DSM-IV, Patient Version[10] The other four studies relied on case-notes. In two studies [11,12] the researchers reviewed patient notes and made the diagnostic decision according to DSM-IV Axis II criteria. One study used the primary psychiatric diagnosis given in discharge summaries from an inpatient psychiatric unit [13] and the other relied on diagnoses in case notes [14]. An array of PD diagnoses were included by authors including antisocial, borderline, paranoid, schizoid, dependent, avoidant, anankastic, and histrionic.

Only four studies contained data for specific diagnoses by ethnic group, these were for borderline PD [10,13], antisocial PD [8.10], and the two combined [10,12]. Only three studies contained prevalence data for PD alone without co-morbidity [9,10,13]. The prevalence data of the other studies included other psychiatric co-morbidity and substance dependence disorders.

### Prevalence

Most studies were concerned with white participants in comparison with black participants. Subgroups of the white ethnic group were not shown in any paper. Five papers failed to provide an ethnic distinction between black sub-groups [7-11]. Five studies (2 of which were scored as high quality) found black populations to have a statistically significant lower prevalence of PD than white populations [11,12,14-16]. One of these studies also determined that Asian populations (from India, Bangladesh and Pakistan) were also less likely to have a PD than white populations [OR 0.1, 95% Confidence Interval (CI) 0.03-0.41, p < 0.05] [12]. However, in contrast to these findings, one large epidemiological survey of a civilian non-institutionalised population determined the weighted prevalence of PD was greater in black populations (16.6%) than white (14.6%) [p < 0.05] [9].

Seven studies were identified as containing raw prevalence data suitable for meta-analysis (additional file 1) [8-14]. All seven studies contained data for black and white participants; in total there were 10356 black participants, and 29954 white participants. The term 'black' includes African-American, African, Afro-Caribbean, and black Other, as used by the original authors. Two studies contained data for Asian participants (n = 1412); in one study [12], Asian referred to those of Indian, Bangladeshi and Pakistani origin; the other study [9] did not define the term. Three studies included data for Hispanic participants [9,10,13] (n = 8815). Three studies were in the UK [11.12.14], and four were in the US [8-10,13]. One study was based in the community [9], one in a prison [10], and five in hospital settings [8,11-14]. The hospital settings included medium security, high security and drug and alcohol addiction units (additional file 1).

### Meta-Analyses

**Figure 2:**
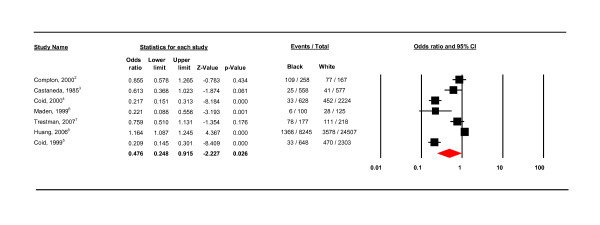
**All studies**.

The initial analyses compared Asians, Hispanic and black groups to whites. There was no significant difference in PD prevalence between Asians and whites (OR O.295 CIs 0.048 - 1.827), or Hispanics and whites (OR 1.155 CIs 0.831 - 1.606). There was, as shown in Figure 2, a significant difference between black and white populations (OR 0.476, CIs 0.248 - 0.915, p = 0.026).

**Figure 3:**
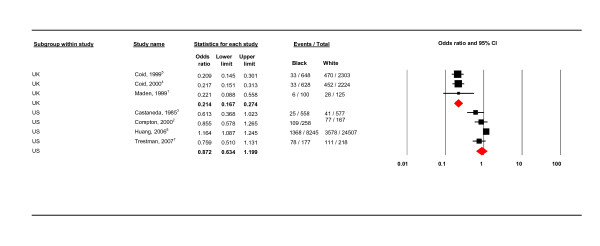
**US and UK studies**.

**Figure 4:**
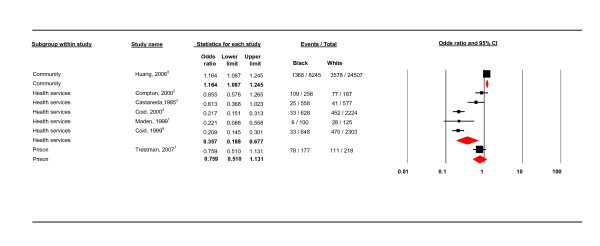
**Study setting**.

**Figure 5:**
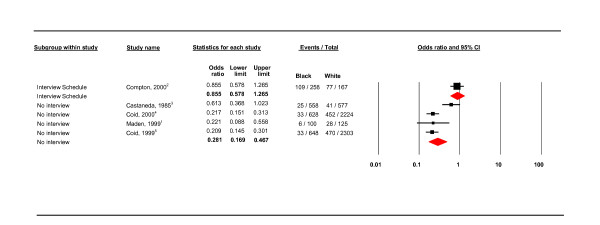
**Health services subgroup; use of interview schedule and no interview schedule**.

**Figure 6:**
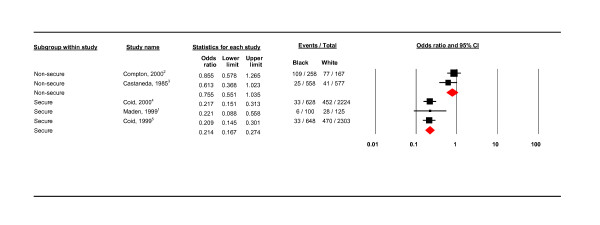
**Secure and non-secure health service study settings**.

**Figure 7:**
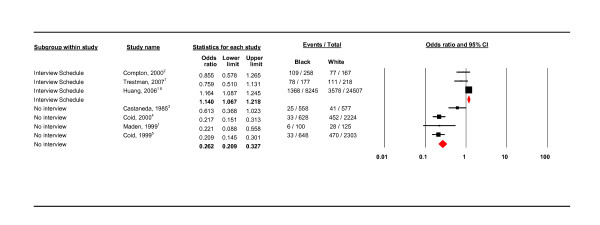
**All studies: interview and no interview use (fixed effects)**.

**Figure 8:**
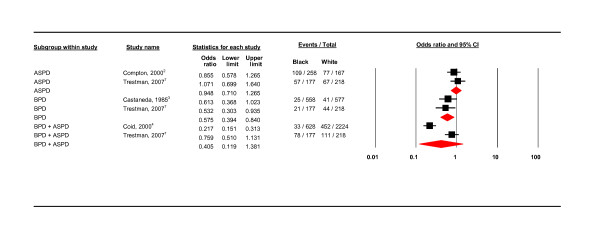
**Diagnosis**.

**Figure 9:**
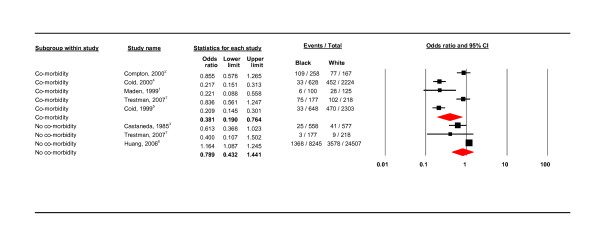
**Co-morbidity and no co-morbidity**.

There was also substantial heterogeneity (I^2 ^= 96.527). Subsequent analyses of potential sources of heterogeneity examined only black and white population data (see Table 4, Figures 3, 4, 5, 6, 7, 8, 9). The country setting, whether conducted in the US or the UK, proved to be an important source of heterogeneity (see Figure 3). There was no significant difference in the prevalence of PD amongst blacks compared to whites in the US (OR 0.872, CI 0.634-1.199, *I*^2 ^= 74.925). In contrast, there was a significant prevalence difference between black and white subjects in the UK studies (OR 0.214, 95% CI 0.167 - 0.274). The UK studies also showed true homogeneity (*I*^2 ^= 0) as shown in Table 4. There were important differences between the US and UK studies; firstly, two of the UK studies were conducted on the same population in secure settings [11,12] and the third UK study was conducted in a similar secure hospital setting [14]. The UK studies also used only case notes whilst the US studies used both interview schedules and case notes (discussed below).

Figure 4 shows that, in a comparison of three service settings (community, hospital and prison), black groups compared to white groups were least likely to have a PD in hospital settings (OR 0.357, CIs 0.188 - 0.677; 89.919) and most likely in community setting (OR 1.164, CIs 1.087 - 1.245). Of the studies in hospital settings, black patients were less likely to have PD in the secure compared to non-secure settings (Figure 6); the three secure setting studies were the three UK studies.

Further meta-regression analysis of the hospital subgroup compared the use of an interview schedule and case-notes diagnoses. Where only case notes were used, the odds ratio was reduced from 0.357 to 0.281 (CI 0.169 - 0.467) (see Figure 5) and heterogeneity was reduced to *I*^2 ^= 77.274.

#### Use of interview schedule

The use of an interview schedule was found to be a source of heterogeneity (see Table 4). The pooled estimate for studies using an interview schedule showed, with a fixed effects model (as *I*^2 ^= 68.815), that the black group was in fact more likely to have a PD than the white group (OR 1.140, 95% CI 1.067 - 1.218; see Figure 7). In contrast, studies not using an interview schedule found the black group to be significantly less likely to have a PD than the white group (OR 0.281, 95% CI 0.169 - 0.467 *I*^2 ^= 77.274; see Figure 5). The interview schedule subgroup were all US studies, the non-interview subgroup included one US study and three UK studies.

#### Diagnosis

Only borderline personality disorder showed a significant prevalence difference between black and white groups (OR 0.575, 95% CI 0.394 - 0.840; *I*^2 ^= 0). These two studies [10,13] were also similar as both were undertaken in the US and used interview schedules. There was also homogeneity (*I*^2 ^= 0) between the two antisocial PD studies but no significant difference between black and white groups in having this diagnosis; these studies were both in the US but used different interview schedules [8,10]. See Figure 8.

#### Co-morbidity

Two of the studies refer to co-morbid drug misuse and dependence but did not specify other diagnoses [12,14]. Compton included co-morbidity with illicit substance misuse and dependence (alcohol and drugs). Trestman included co-morbidity with psychotic, affective, and anxiety disorders and PTSD with cluster A,B,C personality disorders [10]. Coid listed many associations between different PD labels (ASPD + substance misuse, organic brain syndromes; BPD + depression, mania, substance misuse; paranoid PD + drug dependence and psychotic episodes) [11]. In the presence of co-morbidity, black groups were significantly less likely to have a PD diagnosis than white groups (OR 0.381, 95% CI 0.190 - 0.764; *I*^2 ^= 92.288;. See Figure 9). As reflected by the high level of heterogeneity, the co-morbidity sub-group contained mixed studies in terms of setting and use of interview schedule. Where there was no co-morbidity, there was no significant difference between black and white groups (OR 0.789, 95% CI 0.432 - 1.441; *I*^2 ^= 76.081).

### Aetiology

The review found that the aetiology of PDs was the least common subject of research. One study highlighted that Hispanic populations have higher rates of intense anger and affective instability compared to white populations, but these may be manifestations of PD rather than aetiological factors [17]. Several hypotheses about aetiology were found in the publications. It was suggested that certain groups may possess characteristics of particular PDs, migrating ethnicities may find it difficult to adjust, and that higher social classes have lower incidences of PD.

### Treatment

Three of the five high quality scored studies considered race/ethnicity with regards to the treatment of PD [15,18,19]. They determined that more white patients with PD received treatment than black patients. One of these studies comprehensively evaluated types of treatment utilisation by patients with PD and concluded that black and Hispanic patients received a significantly narrower range of psychiatric treatments in spite of having higher rates of severe PD [19]. This was true for outpatient and inpatient psychosocial treatments and psychotropic medications (p < 0.0206 and p < 0.0001 respectively).

In the one RCT identified by the search strategy, which compared community services and conventional hospital-based services for PD, the majority of patients were white (63%)[16] This study determined that those with PD showed greater improvement when treated in the hospital-based setting [16].

## Discussion

### PD diagnosis and ethnicity

The meta-analysis of seven studies determined that overall there was a small but significantly lower prevalence of PD amongst black as compared to white populations. This finding concurred with that of two of the fifteen studies which could not be included in the meta-analysis due to lack of raw data [15,16]. There was no significant difference in prevalence between Asian and white populations, however, only two studies contained this data and it is unlikely that the term 'Asian' connoted comparable populations. The meta-analysis of three studies of Hispanic and white populations showed that Hispanics were more likely to be diagnosed with a PD, however this was not statistically significant.

Where the type of personality disorder was specified, the majority of studies investigated borderline or anti-social personality disorders. Major sources of heterogeneity leading to lower prevalence estimates were the country in which the study was undertaken (US or UK), whether interview diagnoses were made rather than clinical diagnoses, the specific diagnosis of borderline PD versus others, more secure settings and patients with co-morbid disorders. These methodological differences may account for the findings, however, if case note diagnoses are associated with a lower prevalence, this means that the routine care of black patients is likely to overlook PD diagnoses, particularly if they have associated co-morbidity. A recent study using interview diagnoses in the UK investigating prevalence and correlates of PD in provides support for there being no prevalence differences between non-white and white populations [20].

The meta-regression suggests a lower prevalence of PD or that PD is overlooked in more secure settings and in inpatient settings, where acute care is required to manage high risks. If a real difference between settings were to be found using the same methods, then questions about pathways into care and racial bias in diagnostic labelling might be asked. Similarly, the finding of a lower risk of borderline disorder is likely to reflect the differential effects of clinical and case-note diagnoses rather than interview schedules in these studies. However, these findings need replication and the development of case registers from which sufficient numbers of subjects might be gathered to test for these interactions in a more systematic and empirical manner.

### Aetiology

Very little scientific knowledge on the aetiology of PD has been collated [21]. One study highlighted that Hispanics were found to be more intense and angry than whites [17], and another determined that those from ethnic minorities (mostly African Caribbean) and those in higher social classes had a lower incidence of PD [16]. Although there are studies of higher and lower risk in specific demographic and ethnic groups [16,17,21,22], few studies investigate aetiological theories. For example, Chavira et al. investigated whether some ethnic groups had increased vulnerability [17]. Iwamasa et al. proposed that specific ethnic groups were vulnerable to particular PDs [22] rather than all PDs. Castaneda and Franco contend that certain migrating groups may find it difficult to adjust and this is a factor in the development of PD [13]. If prevalence differences are genuine, then identification of different factors across ethnic groups may help in the design of studies to better understand determinants of PD.

### Treatment

Difference in prevalence rates (inpatient and prisoner samples) may be attributed to the differences in help-seeking behaviour by ethnic group and differential effect of 'gate keeping' processes [8,12,15,16,18,19]. Ethnic minority populations may not receive specialist care for PD, in contrast to schizophrenia where black people are over-represented in specialist care, including forensic settings. In the two studies with the highest quality scores, more white than black patients were treated for PD, yet the difference in prevalence did correspond to the lower number of black people hospitalised [15,18], suggesting again the operation of pathway filters that diminish entry into specialist care for black people with PD. Furthermore, in the only study of treatment utilisation, PD and ethnicity, black patients received a significantly narrower range of treatments compared to white patients [19]. Alternatively, more access to treatment may not equate to more effective treatment of PD. For example, variations in compulsory admission to hospital may reflect treatment needs or selection to treatments that appear likely to benefit patients [12]. Bender et al. suggested that non-white patients may have received a narrower range of treatments due to differences in ethnic metabolisms, or the prescribing habits of different mental health workers[19] but few studies replicate these findings or propose an overall theoretical framework within which research studies can lead to improved clinical practice. However, the one RCT concluded that regardless of ethnicity, patients with PD showed greater improvement in social functioning when treated in hospital as opposed to the community; this is the only study comparing different psychiatric venues for the treatments of PD [16].

### Strengths and limitations

The main limitation is the small number of studies included in the meta-analysis. There was also substantial heterogeneity amongst these studies the main sources of which appeared to be study methods, setting and design. However, we stress the importance of this research as innovative. To our knowledge, this is the only review that considers existing research on PD prevalence, aetiology and treatment in relation to race and ethnicity. This research forms part of a larger project of continuing research that will look at specific PDs in relation to race and ethnicity as well as developing and reviewing PD policy involving further research and a panel of experts in the field. At present, we suggest that policy should highlight the need for clinicians to be more culturally aware, and that differences in race and ethnicity must be taken into consideration when diagnosing PDs.

## Conclusion

The existing data are sparse. There is a risk that PD is overlooked and not treated in black people with PD. More specific research in different service settings is necessary to investigate pathways to care. There is almost no aetiological and treatment research on more refined cultural and ethnic categories, leaving unexplained the reasons for differences across broad racial groups.

## Competing interests

The authors declare that they have no competing interests.

## Authors' contributions

A.M and K.S.B designed the study. A.M undertook the initial literature search and review. R.H performed the meta-analyses and meta-regression. K.S.B oversaw and supervised the study. All authors contributed to the preparation of the manuscript and read and approved the final version.

## Pre-publication history

The pre-publication history for this paper can be accessed here:

http://www.biomedcentral.com/1471-244X/10/33/prepub

## Supplementary Material

Additional file 1 **The meta-analysis studies**. Details of the main features of the studies used in the meta-analyses. Click here for file
