# Supplementary material for: A systematic review of personality disorder, race and ethnicity: prevalence, aetiology and treatment
Source: BMC Psychiatry. 2010 May 11;10:33. doi: 10.1186/1471-244X-10-33 (PMC2882360; doi:10.1186/1471-244X-10-33)
Supplement: Additional file 1 — The meta-analysis studies. Details of the main features of the studies used in the meta-analyses. [file 1471-244X-10-33-S1.DOC]

### Additional file 1: The meta-analysis studies

| ***Study*** | ***Country*** | ***Study setting*** | ***Method of diagnosis*** | ***Interview schedule*** | ***PD diagnoses used*** | ***Co-morbidity*** | ***Total Number of participants*** | | | |
| --- | --- | --- | --- | --- | --- | --- | --- | --- | --- | --- |
|  |  |  |  |  |  |  | Black | White | Asian | Hispanic |
| Huang, 2006^8^ | US | Community | Single face-to-face interviews by non-clinicians | AUDADIS-IV | No breakdown. PD includes avoidant, dependent, obsessive-compulsive, paranoid, schizoid, histrionic, antisocial | No | 8245 | 24507 | 1332 | 8303 |
| Coid, 1999^5^ | UK | Secure forensic psychiatry service | Diagnosis made by clinical researcher reviewing case notes | - | No breakdown.  PD includes: antisocial, borderline, paranoid, schizoid, dependent PD | Present. Axis-I and Axis-II disorders | 648 | 2303 | - | - |
| Coid, 2000^4^ | UK | Secure forensic psychiatry service | Diagnosis made by clinical researcher reviewing case notes | - | No breakdown.  PD includes: antisocial, borderline, paranoid, schizoid, dependent PD | Present. Axis-I and Axis-II disorders | 628 | 2224 | 80 | - |
| Maden, 1999^1^ | UK | Medium secure psychiatric units | Diagnosis taken from case notes. | - | Unspecified | Present | 100 | 125 | - | - |
| Castaneda, 1985^3^ | US | Municipal Hospital | Primary psychiatric diagnosis upon discharge obtained from case notes | - | Borderline PD | No | 558 | 577 | - | 402 |
| Compton, 2000^2^ | US | Drug treatment programmes:  both inpatient and outpatient programmes and one residential recovery shelter for women | Two face-to-face interviews conducted by non-clinicians | NIMH Diagnostic Interview Schedule Version III-R | Anti social PD | Drug and alcohol disorders and other psychiatric illnesses | 258 | 167 | - | - |
| Trestman, 2007^7^ | US | Prison inmates | Face to face interview (clinicians and non-clinician interviewers) | SCID-II and SCID-P | Anti-social PD and borderline PD | Data given for both co-morbidity and non co-morbidity PD | 177 | 218 | - | 110 |
